# Just when I thought I was out, they pull me back in: the older physician in the COVID-19 pandemic

**DOI:** 10.1017/S1041610220000599

**Published:** 2020-04-15

**Authors:** Carmelle Peisah, Peter Hockey, Susan Mary Benbow, Betsy Williams

**Affiliations:** 1 University of New South Wales, Australia; 2 University of Sydney, Australia; 3 Capacity Australia, Australia; 4 Western Sydney Local Health District, Australia; 5 Centre for Ageing Studies, University of Chester, UK; 6 Director, Older Mind Matters Ltd, UK; 7 Department of Psychiatry, School of Medicine, University of Kansas, US; 8 Professional Renewal Center ®, Lawrence, KS, US

## Introduction

A call for action to come back and join the fight against COVID-19 has been issued to retired doctors around the world (CBS News, 2020). Many have responded to this call and have returned, or will return to the workforce. Conversely, some late-career doctors who are in practice are anxious about their risk and are understandably choosing now to retire. In this brief perspective, we explore the challenges of the bidirectional movement of older doctors returning to and exiting from the profession at this time of unprecedented stress, to inform solutions, and suggestions for the future.

## Returning to the profession

Regulatory agencies around the world have responded to the need to improve medical workforce capacity in dealing with the COVID-19 pandemic. In the United States, a growing number of American states have taken measures to waive or expedite licensure for inactive or retired medical licensees (FSMB, 2020). A range of models has been developed. These vary according to jurisdiction and are evolving on an ongoing basis. While not all regulatory agencies initially favored relicensing, with the fluidity and urgency of the situation more states are easing requirements for licensure at both the early- and late-career stages (FSMB, 2020).

A diverse range of measures has been implemented to recruit retired practitioners including pro-bono license reinstatement without usual mandatory continuing medical education to those with variable recency of practice ranging from 1 year ago to ever practising. A number of states have implemented restrictions in order to regulate this reentry into the workforce. Models include retired professionals working under a delegation of service agreement with an actively licensed supervising professional and time-limited licenses related to the pandemic. Some states have restricted relicensing to those whose former license was active and in good standing or exempted those with pending complaints, investigations, consent orders, board orders, or pending disciplinary hearings (FSMB, 2020).

Responses of regulatory agencies in other countries are based on similar principles, albeit simplified by being unitary and national. Similar safeguards have been put in place with temporary registration, relative recency of practice, and exemption of those previously sanctioned. The Australian Health Practitioner Regulation Agency (AHPRA) and National Boards (2020) have announced a new short-term pandemic subregister to fast track the return to the workforce of experienced and qualified health practitioners. This subregister will enable doctors who previously held general or specialist registration and left the register or moved to nonpractising registration in the past 3 years to return to practice. Only those who are properly qualified, competent, and suitable will be returned to the register.

In the United Kingdom, under section 18a of the Medical Act (1983), the Government can in an emergency ask the General Medical Council (GMC) to grant temporary registration to doctors who are not currently in practice. That request was made, and the GMC has responded by giving temporary registration to doctors who: (i) left the register or gave up their licence to practise within the last 3 years; (ii) have a UK address; and (iii) do not have any outstanding complaints, sanctions, or conditions on their practice. As of March 27, 2020, the GMC granted temporary registration to 11,856 doctors who had left the profession.

It is not surprising that retired physicians have responded to the call to service and have embraced the opportunity to help. There are many potential factors that might drive retired physicians to do so. Long studied, physician response to retirement is varied, ranging from loss of role, status, self-image, and fulfilment to high levels of life satisfaction imparted by greater opportunities, freedom and relief from exhaustion, the weight of responsibility, and the macro-demands of health systems (Gokce- Kutsal *et al*., 2004; Guerriero Austrom *et al*., 2003; Sadavoy, 1994; Virshup and Coombs, 1993). It is not surprising that some physicians await the call to return from the “afterlife” of retirement, so called because of the sense of “stripped down status” and “living incognito” (Loxterkamp, 2018).

There too exists a risk that retired doctors might feel compelled to volunteer – with attendant feelings of anxiety, shame, and loss of self-worth should they consciously and rationally decide that reentry into the profession is not right for them or their potential patients. As well, some may find it hard to extricate themselves having responded to the call. Although many of the authorities have imposed time-based restrictions on licensure related to the pandemic, no authorities have provided regulations or guidance as to when or how late-career physicians could otherwise terminate their service.

The concept of job-embedded sacrifice influencing psychological attachment and commitment to an organization (Coetzee *et al.*, 2019) has particular relevance to physicians. Physicians are very familiar with the notion of sacrifice (Matz, 1998) and risk to one’s own health is seen as part of the role (Klitzman, 2008) making it easy to get lost in the current zeitgeist where metaphors of war, martyrdom, and heroes abound. Physicians tend to view what they do as more than an occupation, rather as their identity (Sadavoy, 1994). Various codes, for example, the World Medical Association Declaration of Geneva or the Physician’s Oath reinforce the notion of a lifelong commitment, “I solemnly pledge to dedicate my life to the service of humanity.”

If a late-career physician opts to retire or declines the call to return, will this be perceived as a deviation from the Physicians Oath? We suggest not because more grounded ethical imperatives are now at stake here. It is well established that older people, including late-career physicians, are more vulnerable to the coronavirus infection. This is compounded by a shortage of personal protective equipment (PPE) (DelMonico, 2020). If retired physicians are recruited to the frontline, potential harms to the physicians and increased burden on and cost to the health system may outweigh any benefits to staff reserve and workforce.

A further ethical issue is the potential risk of compromising the quality of medical care provided to patients if retired physicians lack necessary competencies to practise. As outlined above, we note that in the scramble to recruit, some states have waived requirements for mandatory continuing education. It is unclear how regulatory authorities will judge retired physicians’ competency to provide clinical care or what precautionary measures will be taken to guarantee compliance with updated management guidelines.

Some have advocated for retired doctors to retain a foothold in medicine (Gokce-Kutsal *et al.*, 2004). This has traditionally meant teaching, management, research, and mentoring, roles often dismissed as “cherry-picking” (Gordon, 2018). Nobody could have anticipated that a return to work might mean dealing with a pandemic and putting yourself at risk.

## Exiting the profession

Why would not an older doctor feel anxious working at this time? Notwithstanding the paucity of any evidence pertaining to older doctors specifically in this or any other epidemic, adverse psychosocial effects on all health care workers are undisputed (Lai *et al*., 2020; Nickell *et al.*, 2004). A cross-sectional survey of 1257 health care workers (39.2% physicians) from 34 hospitals in China from January 29, 2020, to February 3, 2020, found that more than 70% respondents reported psychological distress and a significant proportion experienced depression (50.4%), anxiety (44.6%), and insomnia (34.0%) (Lai *et al.*, 2020). Notwithstanding the fact that fewer than 20% of this sample were aged over 40, the study demonstrates the impact of frontline positions which were associated with more distress and psychiatric symptoms than second-line positions. As with studies from other epidemics such as the SARS outbreak of 2003, risk factors for distress include being a nurse or female (Nickell *et al.*, 2004), although it is likely that such results were confounded by reporting variables with nurses and females more likely to admit distress. This is an important issue for doctors unaccustomed to their lack of omnipotence when it comes to mental health (Henderson *et al.*, 2012), potentially an obstacle for doctors resisting the urge to retire from or return to the workforce.

Compounding this universally experienced psychological response to extraordinary life circumstances (Maunder *et al.*, 2003) is the issue of the physical and cardiovascular risk factors of the older doctor (Pérez *et al.*, 2010) that may render them more vulnerable to the virus, in addition to their age per se.

Pre-COVID, the “stay and go factors” of retirement decision making were driven by a combination of biopsychosocial, macro-related, job, and individual factors (Cleland *et al.*, 2020; Peisah, 2016; Wijeratne *et al.*, 2017). While the “stay factors” may be enhanced by pitching in, a sense of purpose and accolades from the community which extend to special concessions offered to essential services, the “go factors” need not be enumerated. Particular specialities such as emergency medicine (Shin *et al.*, 2018) and intensive care (Skowronski and Peisah, 2012) may be particularly vulnerable to burnout and exhaustion.

Psychiatrists are also currently carrying enormous burden. Increased demands for mental health services have been precipitated by exacerbations of existing mental health conditions and new presentations triggered by the pandemic and the social restrictions in place. This compounds the complexity of delivering community psychiatric outreach and inpatient treatment (including to those detained under mental health legislation and those who are ill due to COVID-19), as well as generic challenges in maintaining staffing to psychiatric services.

## Solutions and suggestions

In galvanising the response to the pandemic, significant concerns have been raised about deploying the existing medical work force outside their specialist field, as has been required in Italy (Paterlini, 2020) into areas of high demand, and what safeguards need to be in place to support this such as supervision, detailed induction, and well-being support (NHS, 2020).

The call to retired doctors has been heralded with the cry that experience and skills are what matter now. However, we advise caution regarding the assumption that an older practitioner who has retired many years ago has either the skills or the experience necessary to practise safely in this current environment, no matter how willing and enthusiastic they are to respond to the call. While it is true that many older clinicians have knowledge, skills, resilience, and wisdom equitable with, and in many cases greater than, younger colleagues, it is equally true that aging is associated with changes in physical and cognitive functioning that affect skills germane to clinical work (Eva, 2002). Increased time since graduation has been correlated with decrements in elements of clinical performance including history taking, physical examination, record keeping, problem solving, and mortality with some complex surgical procedures (Ellison, 2019).

We, like Buerhaus and colleagues (2020) and the American Medical Association (2020), are not suggesting that older physicians should be precluded from providing clinical care, rather we are suggesting that knowing and understanding the risks associated with COVID-19 exposure as well as understanding changes that occur as a function of aging should be considered in determining the optimal roles for late-career physicians. We outline ways this might be achieved, building on the strengths, wisdom, and statesmanship of late-career clinicians while minimizing their risk and exposure:

1. Health care institutions should be mindful of the multiple ethical issues at stake including protection of public from harm and duty of care to patients, not holding doctors hostage to their professional duties and the value of reciprocity enacted by protecting and supporting late-career practitioners (Singer *et al.*, 2003);
2. Deployment of returning doctors should be focused on non-frontline roles in order to free up other staff with the necessary competencies to take on frontline roles (American Medical Association, 2020);
3. Roles including leadership, supervision, consultation, public relations, mentorship, teaching, and research may be particularly appropriate;
4. Roles that involve working remotely (e.g. telephone triage and/or assessment of symptomatic patients) and specialties geared to tele-health provision (e.g. psychiatry) might be better suited for safe deployment of the late-career and returning doctor;
5. Competencies of late-career and returning psychiatrists should be harnessed to support the health and well-being of the existing workforce;
6. Concerns regarding competency might be addressed by using the model adopted by some US states of retired professionals working with an actively licensed supervising professional;
7. Physicians should seek insurance coverage for their return to practice activities; and
8. In the absence of the profession offering guidelines for service termination for returning physicians in the middle of the pandemic, we suggest that physicians take responsibility for their own welfare. Physicians need to decide for themselves where they draw the line (Klitzman, 2008) and have an exit strategy in place should they decide to return to practice. We would encourage physicians, not only to listen to their inner voice but also the voices of those around them – whether they be colleagues or loved ones – who might be expressing their own concerns for the welfare of the physician.

## The future

Just as we are expecting the COVID-19 crisis to impact on the way services are delivered using modern technologies, we should embrace this opportunity to explore how the traditional post-retirement workforce can also contribute in new ways. Not only are many retirees capable and keen to learn new skills, but a lifetime of supporting patients, making decisions and navigating life choices render them ideal mentors and coaches (Bank *et al.*, 2017) for a younger generation. Anecdotal experience of working with recently retired doctors in the UK’s NHS supporting young clinicians undertake complex quality improvement and system change projects has demonstrated real learning (Personal Communication: Kitsell). With a minimum of training in improvement science, they realised that not only were their previous skills very relevant but also recognised the value that this knowledge could have brought to their own working lives. The transfer of this learning, enthusiasm, and recognition of a new skill from an older generation has enabled younger clinicians to step-up and participate in system improvement and change management at an earlier stage of their traditional careers – a remarkable legacy both to individuals and the communities which they serve. We should not lose this opportunity to explore the value that the older medical workforce can bring to individuals and systems which goes far beyond asking older doctors to return to frontline clinical practice.

Protecting and preserving the health care workforce is currently a priority. In achieving this, hospitals and other care delivery organizations, including state and local health departments, must give careful consideration to older physicians (Buerhaus *et al.*, 2020). We suggest that there exists a duty of care to both those staying in the profession and those reentering the profession. For those returning to the workforce, we advise cherry-picking what is manageable while still prioritising self-care. Perhaps a foothold back in is ample.

Concluding on an optimistic note, emerging from the crucible of the coronavirus we may forge new-found ways of fostering both the well-being and contributions of our treasured late-career and retired practitioners into the future.
